# “Brain MR spectroscopy in autism spectrum disorder—the GABA excitatory/inhibitory imbalance theory revisited”

**DOI:** 10.3389/fnhum.2015.00365

**Published:** 2015-06-22

**Authors:** Maiken K. Brix, Lars Ersland, Kenneth Hugdahl, Renate Grüner, Maj-Britt Posserud, Åsa Hammar, Alexander R. Craven, Ralph Noeske, C. John Evans, Hanne B. Walker, Tore Midtvedt, Mona K. Beyer

**Affiliations:** ^1^Department of Radiology, Haukeland University HospitalBergen, Norway; ^2^Department of Clinical Medicine (K1), University of BergenBergen, Norway; ^3^Department of Clinical Engineering, Haukeland University HospitalBergen, Norway; ^4^NORMENT – KG Jebsen Center for Mental Disorders Research, University of BergenBergen, Norway; ^5^Department of Biological and Medical Psychology, University of BergenBergen, Norway; ^6^Division of Psychiatry, Haukeland University HospitalBergen, Norway; ^7^Department of Physics and Technology, University of BergenBergen, Norway; ^8^Department of Child and Adolescent Psychiatry, Haukeland University HospitalBergen, Norway; ^9^MR Applications and Workflow Development, GE HealthcareBerlin, Germany; ^10^CUBRIC, School of Psychology/Ysgol Seicoleg, Cardiff University/Prifysgol Caerdydd WalesCardiff, UK; ^11^Faculty of Mathematics and Natural Sciences, University of OsloOslo, Norway; ^12^Department of Microbiology, Tumor and Cell Biology, Karolinska InstituteStockholm, Sweden; ^13^Department of Radiology and Nuclear Medicine, Oslo University HospitalOslo, Norway; ^14^Faculty of Health Sciences, Department of Life Sciences and Health, Oslo and Akershus University College of Applied SciencesOslo, Norway

**Keywords:** ASD, GABA, MRS, MEGA-PRESS, ASSQ

## Abstract

Magnetic resonance spectroscopy (MRS) from voxels placed in the left anterior cingulate cortex (ACC) was measured from 14 boys with Autism Spectrum Disorder (ASD) and 24 gender and age-matched typically developing (TD) control group. Our main aims were to compare the concentration of γ-aminobutyric acid (GABA) between the two groups, and to investigate the relationship between brain metabolites and autism symptom severity in the ASD group. We did find a significant negative correlation in the ASD group between Autism Spectrum Screening Questionnaire (ASSQ) and GABA+/Cr, which may imply that severity of symptoms in ASD is associated with differences in the level of GABA in the brain, supporting the excitatory/inhibitory (E/I) imbalance theory. However we did not find a significant difference between the two groups in GABA levels.

## Introduction

Autism Spectrum Disorder (ASD) is a pervasive developmental disorder characterized by deficits in social communication and social interaction and by restricted, repetitive patterns of behavior, interests or activities. Symptoms must be present in an early developmental period (before 3 years of age), but they do not necessarily become fully manifest until social demands exceed limited capacities (American Psychiatric Association, 2013). The estimated prevalence of ASD in the Norwegian population ranges from 0.44 to 0.87% (Heiervang et al., 2007; Posserud et al., 2010; Surén et al., 2012) with four times as many boys as girls diagnosed with the disorder (Baron-Cohen et al., 2009b). Autism is now called “spectrum disorder” because of the recognition that its manifestation and severity displays great heterogeneity depending on intellectual ability, associated symptoms, possible etiology and developmental level (American Psychiatric Association, 2013). Although there is clearly a genetic basis to ASD, the majority of cases have unknown causes (Abrahams and Geschwind, 2008; Geschwind, 2008). It is, moreover, now widely accepted that ASD is a neurobiological disorder, but specific biological markers are yet to be established (McPheeters et al., 2011; Warren et al., 2011).

Magnetic resonance spectroscopy (MRS) has made it possible to study the concentration of biochemical substances in the healthy and diseased brain (Soares and Law, 2009). By measuring from a volume element (MRS voxel) in specific regions of interest, metabolite concentrations can be estimated due to differences in spectral resonances from the main water peak. Using the PRESS (Point RESolved Spectroscopy) single-voxel spectroscopy sequence (Bottomley, 1987), metabolites such as *N*-acetylaspartate (NAA), glutamate (Glu), glutamine (Gln), myo-inositol (MI), choline (Cho) and creatine (Cr), can be measured. Unfortunately, other important metabolites such as γ-aminobutyric acid (GABA) are not detectable using conventional MRS, due to spectral overlap with more abundant metabolites at 3.02 parts per million (ppm). One way to measure the concentration of GABA is with a spectral editing technique such as BASING (Star-Lack et al., 1997) or MEGA-PRESS (Mescher et al., 1998). A pair of frequency selective inversion pulses within a standard PRESS sequence allows discrimination between overlapping coupled and uncoupled spins. For GABA editing the editing pulses are applied to the C-3 protons of GABA at 1.9 ppm. Due to the spin-spin coupling the C-4 protons of GABA at 3.02 ppm are affected while other metabolite peaks like the strong Cr peak remains unaffected. Subtracting the spectrum from a second acquisition scheme without these editing pulses (or applied symmetric to the water signal, e.g., at 7.5 ppm), will give a difference spectrum without the strong singlet signal from Cr, allowing quantification of the GABA peak at 3.02 ppm. As these GABA protons are also coupled to macromolecules (MM) at 1.7 ppm this peak consists of GABA and an unknown contribution of MM signal and is therefore named GABA+ (GABA+ MM). This is in line with current best practices (Mullins et al., 2014) and hence compatible with the bulk of existing research including studies on ASD (Gaetz et al., 2014; Rojas et al., 2014).

Glutamate is the major excitatory neurotransmitter and GABA is the major inhibitory neurotransmitter in the brain, and probably all areas receive input from both of these neurotransmitters. The balanced interaction between excitatory and inhibitory neurotransmission is tightly regulated (Carlson, 2001) and is essential for controlling cognition, learning, memory and emotional behaviors. Several studies support the idea that imbalance in the glutamate/GABAergic system could be present in a wide range of disorders with quite different clinical appearances, like Downs syndrome, epilepsy, neurofibromatosis and schizophrenia (Ramamoorthi and Lin, 2011).

Lately increasing evidence have emerged suggesting that also ASD may be associated with abnormalities in the glutamate and GABA system including neurotransmitters, receptors and enzymes (Pizzarelli and Cherubini, 2011) often referred to as the excitatory/inhibitory (E/I) imbalance theory. It has been hypothesized that the E/I ratio in the cortex is unusually high, either due to increased glutamate—or because of decreased GABAergic signaling (Rubenstein and Merzenich, 2003). An E/I imbalance might explain the typical ASD symptom of hypersensitivity to sensory stimuli, including aversion to loud noises, tactile stimulation, and bright lights (Kanner, 1943; Baron-Cohen et al., 2009a).

GABAergic dysfunction in ASD has been proposed in animal models (Gogolla et al., 2009; Chao et al., 2010), post mortem studies and *in vivo* human studies; see overview Coghlan et al. (2012). The E/I imbalance hypothesis is also consistent with the observation that rates of epilepsy are higher in the autism population than in the general population (Gillberg and Billstedt, 2000). Another important finding is that gamma oscillations are reduced in ASD patients (Grice et al., 2001; Brown et al., 2005; Wilson et al., 2007). Gamma oscillations a re generated by GABAergic neurons (Pizzarelli and Cherubini, 2011), and are involved in sensory binding and higher cognitive functions (Lisman and Idiart, 1995).

When starting this study, there was, to our knowledge, only one previous study using the MEGA-PRESS sequence to measure the concentration of GABA in the brain of children with ASD compared with normal controls, and our study was designed to further explore the E/I theory. Harada et al. (2011b) reported significantly lower GABA+/NAA and GABA+/Glu ratio in a voxel placed in the left frontal lobe in a population of children with ASD aged 2–11 years compared to a typically developing (TD) control group (*n* = ASD/TD: 12/10). Since then two more studies have been published. Gaetz et al. (2014) had three different spatially localized voxels; in the left motor cortex (*n* = 17/15), left auditory cortex (*n* = 15/11) and the left visual cortex (*n* = 8/10), respectively. They found that the GABA/Cr ratio was significantly reduced in the motor and auditory cortex, but not significantly different in the visual areas in ASD children aged 11.5 ± 2.7 years compared to TD. The last study, Rojas et al. (2014), found reduced GABA/Cr ratio in a voxel placed in the left auditory cortex in children with ASD aged 14 ± 5 years compared with their unaffected siblings (SIB) aged 12 ± 6 years, and TD aged 12 ± 5 years (*n* = ASD/SIB/TD: 17/14/17).

To explore the excitatory/inhibitory imbalance theory our aim was to study children aged 6–13 with ASD without sedation using both PRESS and MEGA-PRESS sequence. Since studies in healthy individuals have shown significantly higher concentrations of GABA, Glutamate + Glutamine (Glx), and Glu in males compared to females (O’Gorman et al., 2011), only boys were included in the current study.

MRS measured metabolite concentrations are normally presented as ratios of water, total Cr, NAA or in the case of GABA, Glx have also been used. The concentrations obtained from MEGA-PRESS use water as an internal concentration reference, meaning that all our GABA+ data are scaled to water. We have chosen to display our results as GABA+ and GABA+/ Cr since there are reports from previous studies that there exist group differences between ASD and TD in both NAA (Aoki et al., 2012) and Glx (Horder et al., 2013). Gaetz et al. (2014) and Rojas et al. (2014) also used GABA+/Cr to present their results.

**Figure 1:**
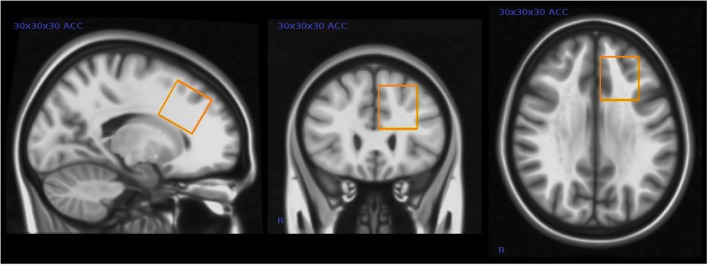
**Placement of magnetic resonance spectroscopy (MRS) voxel in the left anterior cingulate cortex (ACC)**. This figure shows the typical placement of the 3 × 3 × 3 cm MRS voxel in the left ACC.

We assessed GABA and other brain metabolite levels from a voxel in the left anterior cingulate cortex (ACC) using a voxel size of 30 × 30 × 30 mm^3^ (27 ml) see Figure 1.

Although closer to the midline of the brain, this voxel placement partly overlaps the voxel placement in the Harada et al. (2011b) study. This was one of the few locations in the frontal lobe where it was possible to fit the relatively large voxel without including bone or cerebrospinal fluid (CSF) in these children. The dorsal portion of the ACC has been shown to be involved in higher cognition and intellectual functioning (Bush et al., 2000), and other studies have in particular implicated the left ACC in patients with mental disorders (Minzenberg et al., 2009).

All participants were assessed using the Autism Spectrum Screening Questionnaire (ASSQ) and parts of the Wechsler Abbreviated Scale of Intelligence (WASI) as variations in both autism severity and intellectual level may have an impact on metabolite levels. To explore the possible relationship between autism severity and GABA, our GABA+ measurements were correlated with the ASSQ scores (Ehlers et al., 1999). In accordance with the E/I imbalance hypothesis, we hypothesized that we would find a lower concentration of GABA+ and GABA+/Cr ratio in the ASD group compared with the TD group, and that the GABA+ concentration and the GABA+/Cr ratio would correlate negatively with the ASSQ score.

## Methods

### Participants

The boys in the ASD group were recruited through parents groups, patient organizations, newspaper advertisements, from the educational and psychological counseling service in the municipality, and from patients receiving care at a private clinic. The boys in the TD group were recruited from the National Registry. The boys in the ASD group had been independently assessed and diagnosed by child psychiatric outpatient clinical specialists in Norway. Documentation of the clinical assessment leading to an ASD diagnosis was requested and reviewed by a clinical ASD expert (co-author M.P.) for all ASD boys to confirm the ASD diagnosis and ensure that they fulfilled the diagnosis also according to the DSM-5 criteria (American Psychiatric Association, 2013). In addition the parents/legal representatives in the TD group were interviewed using the ASD section of the structured interview Development and Well- Being Assessment (DAWBA) to exclude presence of ASD in the TD group (Goodman et al., 2000). Height and weight of all participants were measured prior to imaging and the parents filled out a screening questionnaire for mental health including the Strengths and Difficulties Questionnaire (SDQ; Goodman, 1997), the ASSQ (Ehlers et al., 1999), items on learning problems, obsessive compulsive disorders, tics and the DSM-IV criteria for attention deficit/hyperactivity disorder (ADHD) and oppositional defiant disorder (ODD), to rule out presence of neuropsychiatric conditions in TD children (Heiervang et al., 2007; Posserud et al., 2009). The ASSQ has been thoroughly validated as measure of ASD and was furthermore used as measure of autism severity (Ehlers et al., 1999; Posserud et al., 2009). The two sub-tests Vocabulary and Matrices Reasoning of the WASI as tests of general intellectual capacity and cognitive skills (Wechsler, 1999) were assessed in both groups by trained psychology students. In addition, information regarding ethnicity, other illnesses, medication, and supplements and diets were noted. Exclusion criteria in both groups were braces (for the MR investigation), genetic abnormalities and prematurity (<36 weeks). Epilepsy, autistic traits and other neuropsychiatric disorders were exclusion criteria in the TD group.

We included boys with epilepsy in our ASD group since it is estimated that 30% of ASD patients also have epilepsy, and subclinical epileptiform activity is recorded on scalp EEG in up to 85% of children with ASD (Gillberg and Billstedt, 2000; Yasuhara, 2010). By excluding children with epilepsy we found that our ASD group would not be representative of children with ASD in general.

In the ASD group, 20 boys were initially recruited from which 14 (mean age 10.2 ± 1.9 years), completed the MRI examination with acceptable PRESS and MEGA-PRESS data as per our quality criteria described in the later “MRS data analysis” section. Three boys in the ASD group did not complete the WASI tests but they were nevertheless included for MRS analyses.

TD boys were recruited as control participants. In the TD group, 30 boys were initially recruited. Three of them were excluded prior to the data analyses; one had a large arachnoid cyst, and two scored above the 90-percentile relative to the population norm from the Bergen Child Study (Heiervang et al., 2007) on the screening questionnaires. Three TD boys did not complete the MR examination. This resulted in a total of 24 boys, mean age 10.2 ± 1.8 years in the TD group with acceptable PRESS data, while MEGA-PRESS data was acceptable in 21.

### Ethical Approval

The study was approved by the Regional Committee for Medical and Health Research Ethics, and was conducted in accordance with the Declaration of Helsinki. Written informed consent was obtained from the children’s parents/legal representatives.

### Magnetic Resonance Imaging (MRI)

Brain MRI scans were acquired using a 3T GE Signa HDxt MR scanner (GE, Milwaukee, USA) equipped with an 8-channel head coil. The following MR protocol was used for acquisition of brain metabolites:

1. PRESS sequence with TE = 35 ms and TR = 1500 ms, 128 averages giving a total acquisition time (TA) of 3:48 min.
2. MEGA-PRESS with TE = 68 ms and TR = 1500 ms, 128 averages each for edited and unedited parts giving TA = 7 min. The somewhat low number of averages was necessary to minimize scan time with a difficult subject demographic.

GABA-editing was achieved with 16 ms 180° Gaussian editing pulses applied at 1.9 ppm and 7.5 ppm with alternating acquisition, giving the “edit-on” and “edit-off” spectra with the GABA spectrum obtained by taking the difference between the two acquisitions. Water reference lines were acquired as a part of the acquisition both for PRESS and MEGA-PRESS.

The MR scanning protocol also included a 3D T1 weighted anatomical scan (number of slices = 192, slice thickness = 1.0 mm, repetition time (TR) = 7.8 ms, echo time (TE) = 2.95 ms, field of view = 260 × 260 mm^2^ flip angle = 14°, matrix = 256 × 256) for anatomical imaging and positioning of the MRS voxel. The T1-images from the SPGR sequence were reformatted from sagittal to oblique axial slice direction with slices positioned parallel to the line connecting the lowest edge of the splenium and the rostrum sections of the corpus callosum. The ACC voxel was then positioned on the left side near the midline of the brain between the genu and middle section of the corpus callosum with the lower voxel edge tangential with the upper border of corpus callosum, to avoid CSF, bone and fat contamination.

### MRS Data Analysis

Water-scaled metabolite concentrations from the PRESS and the MEGA-PRESS data were analyzed with the LCModel analysis software version 6.3-1H (Provencher, 1993). A simulated MEGA-PRESS basis set and constrained baseline were used in the fit. For valid PRESS results the standard deviation was required to be %SD < 20% (Cramér-Rao lower bounds/CRLB) and %SD < 20% for the GABA values in the MEGA-PRESS data (Tables 1, 2).

**Table 1:** **SNR (signal to noise ratio) and linewidth expressed as FWHM (Full width at half maximum) in ppm**.

MEGA-PRESS	ASD (*n* = 14)	TD (*n* = 21)	*P*-value
SNR	20.7 ± 4.1/20.5	19.9 ± 2.6/20.0	0.41
FWHM	0.044 ± 0.014/0.038	0.038 ± 0.010/0.033	0.26
**PRESS**	**ASD (*n* = 14)**	**TD (*n* = 24)**	***P*-value**
SNR	26.6 ± 5.3/28.0	28.7 ± 2.6/29.0	0.39
FWHM	0.047 ± 0.015/0.046	0.044 ± 0.009/0.048	0.82

**Table 2:** **% CRLB (Cramer-Rao Lower Bound) for selected metabolites**.

MEGA-PRESS	ASD (*n* = 14)	TD (*n* = 21)	*P*-value
GABA+	0.07 ± 0.015/0.07	0.07 ± 0.011/0.06	0.70
**PRESS**	**ASD (*n* = 14)**	**TD (*n* = 24)**	***P*-value**
Cr	0.02 ± 0.006/0.02	0.02 ± 0.004/0.02	0.94
NAA	0.02 ± 0.005/0.02	0.02 ± 0.003/0.02	0.25
MI	0.04 ± 0.007/0.03	0.03 ± 0.006/0.03	0.75
Cho	0.02 ± 0.004/0.02	0.02 ± 0.002/0.02	0.39
*Glx*	0.04 ± 0.008/0.04	0.04 ± 0.006/0.04	0.99

**Figure 2:**
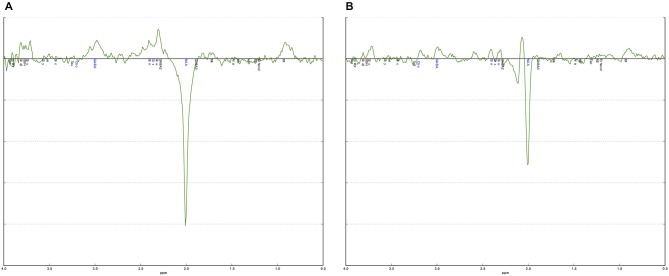
**Examples of a good and a poor quality MEGA-PRESS spectrum. (A)** The MEGA-PRESS spectrum shows a clearly visible GABA+ peak at 3.0 ppm. The difference between the data and the fitted curve, the residual, is small indicating a good fit. **(B)** The MEGA-PRESS spectrum is noisy with no clear GABA+ peak at 3.0 ppm. The residual is also large indicating a poor fit.

The curve fitting of the GABA peak at 3.02 ppm was visually inspected in each individual. Additional validation was performed using an in-house quality-assurance script implemented in Python.^1^ This script validates individual spectra against a typical (group average) spectrum for the region and identifies any aberrations in shape of the spectra or features in the residuals—as would result from artifacts or poor fitting. It additionally checks quantitative metrics such as linewidth, signal-to-noise, and CRLB across the fit. This process is intended to guide and to complement regular visual inspection (Figure 2).

### Statistics

Demographic data, ASSQ, intellectual ability (WASI) and MRS data were analyzed using SPSS (version 12.01) (IBM Corp. USA) statistical analysis software package. MRS measurements were non-normally distributed, even after log transformation, and results were thus analyzed using the non-parametric Mann Whitney U-test and Skipped Pearson and Spearman correlation (Pernet et al., 2013) with the level of significance being *p* ≤ 0.05. The GABA+/Cr ratio was normally distributed and the Students *t*-test was therefore applied.

### Quantifying Tissue Composition

GABA tissue concentration has been demonstrated to differ between gray matter (GM) and white matter (WM; Petroff et al., 1988). The segmentation of GM, WM and CSF within the MRS voxel was carried out on the 3D-SPGR images. Segmentation was performed using in-house scripts based on SPM’s unified segmentation and normalization functionality.^2^ The same software was also used to calculate total GM and WM volume and total brain volume.

## Results

### Medication

Five of the 14 boys in the ASD group used psychotropic medication; one was medicated with lamotrigine (antiepilepticum), two with metylphenidate (ADHD medication), one with aripriprazol (antipsychotic medication) and one with metylphenidate and levetiracetam (antiepilepticum). A further two used melatonin. None of the participants in the TD group were on medication.

### Autism Severity

All TD boys scored below the screening cut-off for ASD on the ASSQ, and all the ASD boys scored higher than all the TD boys (mean ASSQ score 23.5 in the ASD group vs. 1.5 in the TD group, *p* < 0.001).

### WASI Two Subtest Format

The results for the WASI total score and scores for the two subtests are shown in Table 3.

**Table 3:** **WASI IQ scores**.

	ASD (*n* = 11)	TD (*n* = 24)	*P*-value
WASI, total score	97.6 ± 19.6/95.0	108.3 ± 14.3/107.0	0.11
Raw score Vocabulary	30.8 ± 15.4/34.0	40.4 ± 9.4/41.5	0.04
Raw score Matrix Reasoning	19.7 ± 8.3/23.0	25.8 ± 9.3/26.5	0.07

*Data are presented as mean ± standard deviation/median value*.

As can be seen in Table 1, the ASD group scored below the TD group on all three WASI variables. The difference is significant for the Vocabulary scores, and approaches significance for the Matrix Reasoning scores (*p* = 0.07). However, the total score including both subtests was not significantly different between groups and both groups were within normal range of intellectual capacity.

### MRS Results

**Figure 3:**
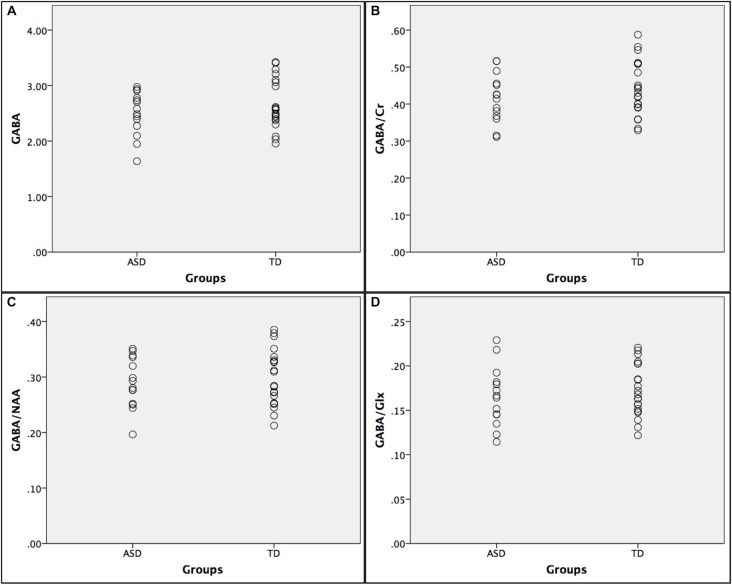
**Scatter plots showing the distribution of GABA+ ratios in the autism spectrumdisorder (ASD) and typically developing (TD) groups. (A)** GABA+; **(B)** GABA+/Cr; **(C)** GABA+/NAA; and **(D)** GABA+/Glx.

Below is a summary of findings for both the PRESS and MEGA-PRESS sequences in the two groups. Table 4 there were no statistically significant differences between the two groups (ASD/TD) in GABA+ and GABA+/Cr levels. For comparison with previous studies, analyses were also performed with GABA+/Glx and GABA+/NAA, but no statistically significant differences between the two groups were found. See also scatter plots for the distribution of GABA+ and GABA+/Cr in the two groups (Figure 3).

**Table 4:** **The summarized results for the quantization of relevant metabolites from the MEGA-PRESS and PRESS sequences**.

MEGA-PRESS	ASD (*n* = 14)	TD (*n* = 21)	*P*-value
GABA+	2.49 ± 0.40/2.53	2.66 ± 0.45/2.56	0.41
GABA+/Cr*	0.42 ± 0.066/0.42	0.44 ± 0.074/0.43	0.30
**PRESS**	**ASD (*n* = 14)**	**TD (*n* = 24)**	***P*-value**
Cr	6.01 ± 0.54/6.00	6.08 ± 0.39/6.05	0.71
NAA	8.61 ± 0.60/8.66	8.99 ± 0.73/9.08	0.10
MI	5.18 ± 0.87/5.08	4.98 ± 0.54/4.95	0.69
Cho	2.17 ± 0.21/2.16	2.19 ± 0.21/2.18	0.80
*Glx*	15.2 ± 1.58/15.00	15.49 ± 1.17/15.48	0.35

The concentrations obtained from the MEGA-PRESS experiment use water as an internal concentration reference. *Cr from PRESS. Data are presented as mean ± standard deviation/median. Values for metabolites are in Institutional Units.

**Figure 4:**
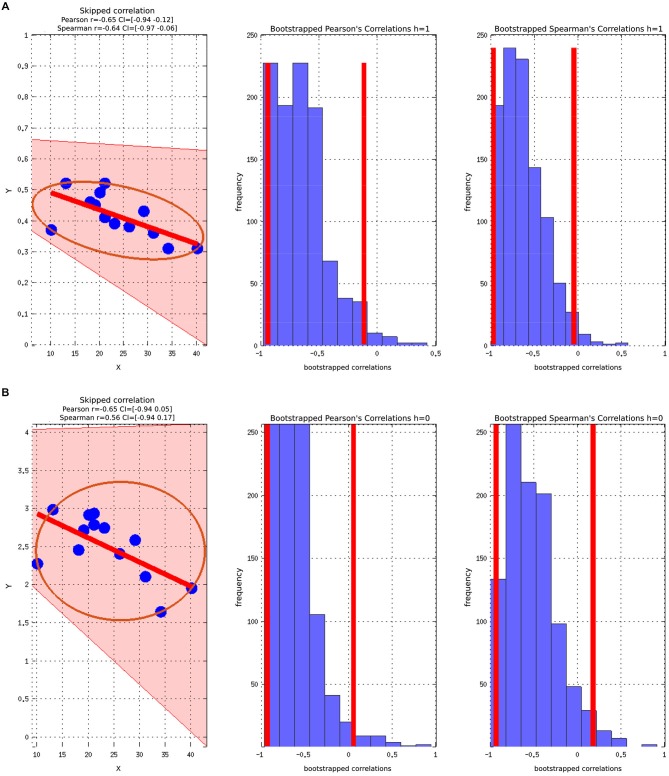
**Skipped Pearson and Spearman correlations with a 95% confidence interval (CI). The figures also include Bootstrapped Pearson and Bootstrapped Spearmans plots. (A)** ASSQ vs. GABA+/Cr; **(B)** ASSQ vs. GABA+.

There was a statistically significant negative correlation between the ASSQ score and the GABA+/Cr concentration in the ASD group, (Figure 4). The significant negative correlation between GABA+ and ASSQ when using the Pearson correlation coefficient did not hold when the skipped Pearson and skipped Spearman test was applied.

The correlations did not survive correcting for multiple comparisons due to the relative low number of participants. Controlling for WASI did not change the strength or direction of the correlation between ASSQ and GABA, but the statistical significance was lost.

ASSQ is a questionnaire instrument applied to measure autism severity with values above the cut off set at 17 indicating ASD. The TD group all had ASSQ scores below 5. Still, correlation analyses were performed between the ASSQ score and GABA+ and the ratios and none of them were statistic significant.

### Tissue Segmentation

There were no group differences in total brain volumes and segmented tissue volumes in the MRS voxel (Table 5). As no systematic differences in tissue composition were observed, these estimates were not considered further in the statistical analysis.

**Table 5:** **Results for the total brain volume ratios measured by tissue segmentation, and results from tissue segmentation in the MRS voxel**.

	ASD	TD	*P*-value
Brain volume Gray	855 ± 72/865	882 ± 75/874	0.50
Brain volume White	405 ± 37/406	418 ± 42/422	0.38
Brain volume total	1261 ± 99/1277	1300 ± 112/1301	0.46
Voxel CSF	5.5 ± 4.7/4.3	3.4 ± 2.6/2.5	0.20
Voxel Gray	44.7 ± 5.6/45.2	45.2 ± 5.2/43.9	0.89
Voxel White	48.7 ± 10.3/53.0	51.3 ± 7.2/53.4	0.39

*Data are presented as mean ± standard deviation/median. All volumes are in ml*.

## Discussion

In our correlation analyses we found significant negative correlations in the ASD group between ASSQ score and GABA+/Cr ratio, indicating that lower GABA+/Cr ratio is associated with increasing autism severity.

This is the first paper, to our knowledge, that has examined autism symptom severity and correlated this with metabolite concentration measured from MRS using a MEGA-PRESS sequence. However, our study supports the MEG-study by Cornew et al. (2012) where they did find an association between oscillatory anomalies, which suggest E/I imbalance, and autism symptom severity. The participants in the Cornew study were 50 children, 27 with ASD and 23 age-matched controls, aged 6–15 years.

Our study did not reveal any group-level differences between the ASD and the TD group regarding the concentration of GABA+ and GABA+/Cr. These results do not fully support all the results from the three previous studies (Harada et al., 2011b; Gaetz et al., 2014; Rojas et al., 2014) that have applied MRS MEGA-PRESS to compare brain GABA+ concentrations in children with ASD.

Harada et al. (2011b) found significantly lower GABA+/NAA and GABA+/Glu ratio in the ASD group, with a voxel placed in the left frontal lobe, partly overlapping with our voxel placement in the left ACC. Gaetz et al. (2014) found a significantly reduced GABA+/Cr ratio in the ASD group in voxels placed in the left motor and auditory cortex, while Rojas reported reduced GABA+/Cr ratio in children with ASD and their unaffected siblings compared to TD controls in a voxel placed in the left auditory cortex (Rojas et al., 2014).

An obvious difference between the three studies is that the voxel placements in our study differed from the others. The ACC region was chosen for voxel placement since the area is implicated in the pathophysiology of ASD due to its crucial role in social cognitive processes (Devinsky et al., 1995; Bush et al., 2000; Amodio and Frith, 2006). The ACC region is also known to be involved/affected in patients with psychiatric disorder including schizophrenia, obsessive–compulsive disorder, depression, post-traumatic stress disorder and ASD (Benes, 1993; Rauch et al., 1994; Devinsky et al., 1995; DeVito et al., 2007; Minzenberg et al., 2009).

Several recent studies implicate the ACC region in ASD, and also connected it to imbalances in GABA/Glu. For example, Naaijen et al. (2015) found aberrant fronto-striatal glutamatergic levels, including the ACC in children with ASD. Similarly, Cochran et al. (2015) found that glutamate-GABA interaction and balance was disturbed in ASD subjects, and that this correlated with social cognition measures. Finally, Hall et al. (2015) used an auditory oddball task that requires complex cognition, and measured both electrophysiology responses and MRS glutamate levels from the ACC and found a significant positive correlation between the two indicating a specific connection between an index of glutamate neurotransmitter function and frontal event-related potential. Thus, the ACC region has been shown to be sensitive for subtle changes in metabolite levels during social cognitive and rest periods.

Both Harada et al. (2011b) and Gaetz et al. (2014) showed that there might be regional differences as they found lower concentration of GABA+ in ASD individuals in some areas of the brain and with no group differences in other areas compared to TD individuals. Further studies are needed to investigate the role of voxel placements for GABA+ measurements.

Another possibility is that there are subgroups in the ASD population where differences in GABA+ or the metabolites used in the GABA+ ratio exist, which may explain the heterogeneity in the ASD phenotype. All four studies on ASD children, referred to in this manuscript, including our study, had small sample sizes. The subjects in the Harada paper had a wide age range (Harada et al., 2011b), and there was no information whether the included subjects were boys or girls, while both boys and girls were included in the Gaetz et al. (2014) and Rojas et al. (2014) paper. In addition the Gaetz paper lacked sufficient information about the recruitment of TD. As pointed out earlier, gender may be a significant factor in predicting GABA+ levels (Harada et al., 2011a; O’Gorman et al., 2011), however other studies have not shown this difference (Puts and Edden, 2012). We need more knowledge on how age and gender influences GABA+ levels and we cannot rule out that such group differences contribute to the previous results.

A strength in our study is that we only included boys, and there were no age differences between the two study groups. Moreover, both groups had total mean IQ scores within normal range. Thus we think our results are representative for boys in this age group. The discrepancy between the groups in IQ with lower verbal IQ scores in the ASD group was expected, since it reflects a clinical characteristic of children with ASD (Mayes and Calhoun, 2003).

Controlling for WASI did not change the strength or direction of the correlation between ASSQ and GABA+, indicating that WASI was not a confounder in our analyses. Thus the variation in performance on WASI (IQ) seems secondary compared to a composite measure of ASD symptoms (ASSQ) to describe the variation GABA+/Cr ratio in the ACC. The lower GABA values were associated with more severe ASD symptoms. More in depth neuropsychiatric tests were not included in this study, therefore it is not possible to evaluate performance of functions typically associated with the ACC region such as executive control, theory of mind etc. While there may be some association between regional GABA+ content and neuropsychological performance, further studies are needed to explore these possible confounding factors.

Use of medication differed between the groups; 5 of 14 boys in the ASD group used psychotropic medication and an additional two used melatonin, vs. no medications in the TD group. GABA inhibition is a well-known target for therapeutic anticonvulsant treatment, but neither of the two antiepileptic drugs lamotrigine and levetiracetam have GABA enhancement as their main effect (Olsen and Avoli, 1997). An MRS study by Kuzniecky et al. (2002) showed a 25% increase in GABA concentration on five healthy volunteers administered with long-term dosing of lamotrigine measured from a voxel in the occipital lobe. The exact mechanism of levetiracetam in treating epilepsy is unknown, and *in vivo* studies of levetiracetam on whole mice brain preparation haven not detected any differences on the overall GABA and glutamate concentration (Sills et al., 1997).

All analyses were performed both with and without the two boys using antiepileptic drug and it had no significant effect on the results for the group level analyses.

Methylphenidate has a dopaminergic effect similar to amphetamine and cocaine by blocking the dopamine and noradrenaline transporters in pre-synapse, thus increasing the availability of these neurotransmitters in the synaptic cleft. GABA can modify the activity of dopaminergic neurons, and psychostimulants (Pierce and Kalivas, 1997) may also influence the activity of GABAergic neurons directly. One study on female rats found that repeated treatment with methylphenidate significantly increased the expression of GAD65 and GAD667 in the prefrontal cortex thus increasing vesicular GABA levels (Freese et al., 2012). There is limited research available on the effects of methylphenidate on GABA levels in humans; therefore a possible impact on GABA levels cannot be excluded.

An 18F-Fluoroflumazenil PET study on 17 patients with schizophrenia found that aripiprazole administration resulted in increased GABA transmission in the prefrontal cortex (Lee et al., 2013).

Melatonin activates the benzodiazepine-GABAA receptor complex with consequent enhancement of GABAergic activity (Niles, 2006).

A common denominator of the four types of psychotropic medication and melatonin is that they may all increase GABA+ levels, and they may thereby potentially mask an underlying group difference in GABA+ level between the ASD and the TD group. When comparing with the three other MEGA-PRESS studies on children with ASD, we found no information regarding epilepsy or usage of psychotropic medication in the Harada et al. (2011b) paper. Gaetz et al. (2014) and Rojas et al. (2014) excluded children with epilepsy from the ASD group. In the Rojas et al. (2014) study five ASD children used psychotropic medication, four SSRI and one an atypical antipsychotic drug. In the Gaetz et al. (2014) study four ASD children used psychotropic medication, two a SSRI, one a “medication to treat mood disorder” and one atypical antipsychotics. Regarding the effect of medication on GABA+/Cr ratio, Rojas et al. (2008), Gaetz et al. (2014) and our own study are comparable.

Most of the children in the Harada et al. (2011b) study were sedated with triclofos sodium, a GABA agonist, which might complicate the interpretation of their results. Reduced Glu and increased GABA have been observed bilaterally in all cortical regions examined following sedation with propofol (Zhang et al., 2009), which is another GABA agonist. It may be that an effect of sedation has influenced the MRS measurements and contributed to their results.

Beyond any real underlying physiological variations, our GABA+ measurements may also be affected by the different MRI acquisition parameters used—in particular, the number of averages. We chose to average over 128 pairs in order to keep the scan time down and thereby minimize any errors due to head movement. Lying completely still in the scanner is challenging for children, and even more so for children with ASD. Both the Harada et al. (2011b) and the Gaetz et al. (2014) paper used 128 averaged pairs in their MR protocols, while in the paper by Rojas they averaged 256 pairs (Rojas et al., 2014), giving twice as many spectral acquisitions and correspondingly improved signal-to-noise, but also increasing the scan time significantly (to about 20 min with their TR of 2500 ms). A recent study from our group (Craven abstract ISMRM 2014) suggests that an average of 256 pairs at TR = 1500 ms is more optimal for measuring GABA+ using MEGA-PRESS.

Applying the MEGA-PRESS GABA-editing MRS sequence for measurement of GABA+ in the human brain has become increasingly of interest during the last couple of years, especially when evaluating neurochemical underpinnings of psychiatric disorders. Even so, the most stable denominator for normalized GABA+ measures, like basic sequence parameters and specific method of spectral quantification, still remains an active area of investigation. Thus, it is a possibility that there exists a difference in GABA+ levels between groups, but that we were not able to measure it. MR parameters, like number of averages, and voxel placement are probably more a factor than the MEGA-PRESS method itself, and with further optimization a more subtle difference may become apparent.

As mentioned in the introduction, ASD patients represent a very heterogeneous group, probably reflecting variability within the autism spectrum with different neurobiological substrates. It is hardly likely that we will find one causal model for ASD. Using different methods, e.g., questionnaires and standardized interviews, to subgroup the ASD population and understand the mechanisms involved may prove vital in future ASD studies, including MRS studies.

## Conclusion

We found a negative correlation between autism severity, as measured by the ASSQ and GABA+/Cr in the ASD group, but no GABA+ or GABA+/Cr level differences between ASD and TD groups. The intricate balance between GABA and glutamate in the brain in ASD is not completely understood. Recent evidence even supports a deviant function of the GABA neurotransmitter in children with ASD with an excitatory rather than an inhibitory effect indicating that absolute levels of GABA and glutamate may not reflect the E/I imbalance in the same way in ASD as in other patient groups (Tyzio et al., 2014).

The present study clearly shows that autism severity must be taken into account to elucidate the E/I hypothesis in autism. Future studies with larger cohorts of ASD subjects in different ages, severity and gender, and in different brain regions are required to reveal the levels of GABA and glutamate and the importance of the balance between them in relation to ASD symptoms.

## Author Contributions

Substantial contribution to the conception or design of the work; or the acquisition, analysis, or interpretation of data for the work; MKB, LE, KH, RG, MBP, ÅH, ARC, RN, CJE, HBW, TM, MKB (Beyer).

Drafting the work or revising it critically for important intellectual content; MKB, LE, KH, RG, MBP, ÅH, ARC, RN, CJE, HBW, TM, MKB (Beyer).

Final approval of the version to be published; MKB, LE, KH, RG, MBP, ÅH, ARC, RN, CJE, HBW, TM, MKB (Beyer).

Agreement to be accountable for all aspects of the work in ensuring that questions related to the accuracy or integrity of any part of the work are appropriately investigated and resolved: MKB, LE, KH, RG, MBP, ÅH, ARC, RN, CJE, HBW, TM, MKB (Beyer).

## Conflict of Interest Statement

The authors declare that the research was conducted in the absence of any commercial or financial relationships that could be construed as a potential conflict of interest.

## Footnotes

^1^https://www.python.org

^2^http://www.fil.ion.ucl.ac.uk/spm/software/spm8/

## Abbreviations

ASD: Autism Spectrum Disorder
MRS: Magnetic Resonance Spectroscopy
ppm: Parts per million
NAA: *N*-acetylaspartate
Glu: Glutamate
Gln: Glutamine
MI: Myo-inositol
Cho: Choline
Cr: Creatine
PCr: Phosphocreatine
GABA: Gamma-aminobutyric acid
MM: Macromolecules
GABA+: GABA including associated MM
E/I: Excitatory/inhibitory
TD: Typically developing
SIB: Unaffected siblings
Glx: Glutamate and glutamine
ACC: Anterior cingulate cortex
ASSQ: Autism Spectrum Screening Questionnaire
WASI: Wechsler Abbreviated Scale of Intelligence
DAWBA: Development and Well-Being Assessment
SDQ: Strengths and Difficulties Questionnaire
ADHD: Attention deficit/hyperactivity disorder
ADD: Oppositional defiant disorder
TR: Repetition time
TE: Echo time
CRLB: Cramér-Rao lower bounds
CSF: Cerebro spinalfluid
Gray: Gray matter
White: White matter.
